# Thermal Infrared Sensing for Near Real-Time Data-Driven Fire Detection and Monitoring Systems

**DOI:** 10.3390/s20236803

**Published:** 2020-11-28

**Authors:** Maria João Sousa, Alexandra Moutinho, Miguel Almeida

**Affiliations:** 1 IDMEC, Instituto Superior Técnico, Universidade de Lisboa, Av. Rovisco Pais, 1, 1049-001 Lisboa, Portugal; alexandra.moutinho@tecnico.ulisboa.pt; 2 ADAI, University of Coimbra, Rua Pedro Hispano, 12, 3030-289 Coimbra, Portugal; miguelalmeida@adai.pt

**Keywords:** thermal infrared cameras, thermal imaging data, wildfire detection, active fire monitoring, early warning systems, unmanned aerial systems

## Abstract

With the increasing interest in leveraging mobile robotics for fire detection and monitoring arises the need to design recognition technology systems for these extreme environments. This work focuses on evaluating the sensing capabilities and image processing pipeline of thermal imaging sensors for fire detection applications, paving the way for the development of autonomous systems for early warning and monitoring of fire events. The contributions of this work are threefold. First, we overview image processing algorithms used in thermal imaging regarding data compression and image enhancement. Second, we present a method for data-driven thermal imaging analysis designed for fire situation awareness in robotic perception. A study is undertaken to test the behavior of the thermal cameras in controlled fire scenarios, followed by an in-depth analysis of the experimental data, which reveals the inner workings of these sensors. Third, we discuss key takeaways for the integration of thermal cameras in robotic perception pipelines for autonomous unmanned aerial vehicle (UAV)-based fire surveillance.

## 1. Introduction

With the emergent effects of climate change, several regions worldwide have been undergoing an increasing number of more intense and devastating wildfire events, as well as extended fire seasons [1,2]. In this context, given the high spatial and temporal uncertainty intrinsic to these phenomena, environment monitoring is determinant for firefighting activities to mitigate the consequences of these events.

Currently, limited areas can be monitored through automatic systems in watchtowers. However, by being installed near the ground—hence, at low altitudes—these systems have several limitations. Since these are widely based on visible range sensors, clouds can be easily confused with smoke and the sunset or reflections can be mistaken by flames, leading to false alarms. Moreover, as the systems depend on the identification of a smoke column, flames can only be detected when the fire has increased in magnitude, thus often preventing early fire detection. Furthermore, solutions based on satellite data have considerable latency, thus also hindering their application for early detection.

Despite the widespread use of aerial means in prevention of and emergency response to forest fires, piloted aircraft require highly trained personnel and are expensive to operate [3,4], limiting the number of vehicles that are used for firefighting and surveillance tasks. However, given that these scenarios require the coverage of extensive areas where the environment is highly dynamic, the availability of aerial means is paramount on tactical and operational levels for situation awareness.

To address this issue, in recent years, there has been active research towards developing systems based on unmanned aerial vehicles (UAVs) for fire detection [5,6] and operational support [7,8,9], but it has intensified lately as a result of the difficulties faced in responding to large-scale wildfires, which has reinforced the need to detect fires in an early stage, as well as to provide near real-time monitoring.

In parallel, the recent advances in robotic perception have opened a path towards autonomous robotics by taking advantage of novel sensors and intelligent systems to enable autonomous navigation and exploration [10]. The breakthroughs in sensor technology and embedded computing have resulted in the progressive decrease in the weight/size ratio and equipment cost while incorporating powerful computing capabilities. These emerging technologies are enabling real-time processing of high-dimensional data, which allows UAVs to be equipped with advanced thermal and optical image sensors as well as computing platforms for on-board data processing.

In this context, the general aim of this investigation is to contribute to the development of UAV-based systems for fire detection and monitoring, namely in what concerns the integration of thermal cameras in robotic perception for these tasks.

The main objective of this study is to provide a comprehensive understanding of the behavior of thermal imaging sensors in fire detection scenarios by relating the sensor response and the image processing employed in this type of device. In that sense, this work explores sensor data from two different thermal cameras by linking the raw sensor data to the mapping functions employed to obtain images encoded in pseudocolor. For this purpose, several fire experiments in controlled conditions were performed in laboratory and field trials covering distinct operating regimes of this type of sensor.

The contributions of this article are threefold: (1) an overview of state-of-the-art image processing algorithms widely used in thermal imaging cameras; (2) an in-depth analysis of the behavior of thermal cameras targeted at fire identification scenarios based on controlled fire experiments; (3) discussion of the implications of the insights exposed and the potential developments in robotic perception towards autonomous UAV-based fire detection and monitoring systems.

Considering the growing availability of low-cost, compact thermal cameras for UAV-borne applications, the uptake of this technology will progressively increase in the near future [11]. As this topic is an active area of research, the relevant insights outlined in this article provide important considerations to guide future research. Hence, also contributing to the practical implementation of thermal-enabled fire detection and monitoring systems, which bring great potential for minimizing the impacts of fire events.

### 1.1. Related Work

The advent of the evolution of remote sensing technologies has led to the continuous improvement of fire hazard identification and risk assessment systems. Over the years, several types of platforms have been explored for these solutions, such as satellites, high-altitude aircraft, and remotely piloted aircraft systems, enabling the assessment of the progression of fire events [12].

Although satellite-based imagery is used by emergency response agencies to monitor large-scale wildfires that burn over extensive periods, the wait interval for a satellite overpass induces a considerable time delay, which prevents its application in time-sensitive fire detection scenarios, such as emergency evacuations or search-and-rescue operations [13]. Despite its value from a strategic standpoint, for tactical and operational decision support, the availability of updated information is crucial. To address this while avoiding the expensive operation costs of piloted aircraft, UAVs are considered as a viable alternative for remote sensing by providing local coverage with high spatial and temporal resolution.

In previous contributions, wildfire detection applications based on airborne systems have explored visible range, thermal, or multispectral technologies [14]. However, since the radiation emitted from a fire is high in the thermal range, there has been significant interest in the use of thermal infrared bands [12]. In that sense, the following review focuses on contributions employing the thermal range.

Thermal infrared cameras provide sensing capabilities suitable for ongoing environmental monitoring by operating in both daylight and in night conditions. Additionally, in contrast to visible range sensors, for which smoke severely affects the ability to detect and track the perimeters of fire fronts, for thermal infrared cameras, the impact of smoke presents lower interference.

Regarding the development of automatic algorithms, several contributions have presented advances towards this objective. On the one hand, works on image processing algorithms have focused on the extraction of image descriptors to obtain signals representing fire instances to detect the presence of a fire by assessing the time-series data [15]. On the other hand, since thermal images adapt according to the context in the field of view of the camera, considering only the brightness information can lead to false alarms. To address this issue, some approaches combine both spatial and temporal features, i.e., brightness, motion, and flicker [16]. In turn, the integration of infrared cameras has been suggested as a way to complement visual range fire surveillance systems so as to harness the advantages of both visual and thermal features and to yield more accurate early fire detection rates [17,18,19]. More recently, an off-line processing fire-tracking algorithm based on edge detection has been proposed to process geo-referenced thermal images previously acquired using an airborne thermal camera [20].

Note, however, that not all the previously mentioned research efforts focus specifically or solely on wildfire detection. In a wider scope, considering fire detection outdoors, authors have recognized the difficulty in applying image processing algorithms in this setting due to a significant rate of false positives caused by external factors, such as weather conditions, sunlight reflections, or saturation of the infrared sensors caused by other heated objects [21]. Given the challenges faced in real contexts, it becomes rather complex to overcome these limitations with ad hoc classical computer vision algorithms.

In alternative to computer vision methods, to improve generalization in dynamic scenarios, intelligent systems approaches have been proposed, namely data-driven models based on feature engineering and fuzzy inference systems [22]. The nonlinear approach has been successfully tested in fire experiments under controlled conditions and validated in highly dynamic environments, such as camping sites.

Most previous approaches were designed for data acquisition purposes and algorithm design, whereas only recently autonomous systems are being developed, as a result of the increased data processing capabilities aboard aerial vehicles.

### 1.2. Proposed Approach

Thermal cameras have a clear potential for wildfire detection and monitoring tasks, but the path towards their integration in robotic perception pipelines, which are essential for autonomous systems, is still rather understudied. To contribute to narrowing this gap, this work conducts a comprehensive study of thermal imaging sensing to extend the understanding of the inner workings of this technology and how it can be leveraged for wildfire surveillance tasks. For that purpose, it is important to identify the main challenges this work addresses in the following.

First, although thermal imaging cameras are increasingly available for a multitude of industrial domains [23], with expanding model ranges and accessible equipment costs, their application is still limited, which can be attributed to two factors. On the one hand, most applications rely on a human-in-the-loop approach, which typically requires specialized training and technical expertise to interpret the image data, but this is generally based on high-level knowledge oriented to the domain of application. On the other hand, as discussed in the literature review, machine vision approaches depend on feature-based approaches derived from image data, which do not take into account the image processing algorithms underlying the output data. However, these abstraction layers hinder the development of automatic algorithms due to the adaptive nonlinear nature that is at the core of the behavior of these systems. Therefore, the knowledge of the underlying processing methods involved in generating the output image is central to understanding how to leverage this technology in a robotic perception framework. In that sense, the first step in this work concerns the overview of state-of-the-art image processing algorithms employed in most commercial off-the-shelf thermal cameras.

Second, the adoption of thermal imaging cameras for wildfire detection scenarios differs considerably from general applications, e.g., industrial inspection or precision agriculture, in the sense that it deals with extreme temperatures. In this context, the importance of quantitative information is less prevalent than that of qualitative data because a fire can be identified by high temperature gradients with respect to ambient conditions, and can thus be detected using the relative temperature differences in the images. To that effect, having radiometric information is not determinant because the intensity levels can translate the relative difference between objects in the scene. Nonetheless, a correct interpretation of the adaptive algorithms is required because the color-encoding schema adapts to the range of measurements in each instance. For these reasons, after covering the processing algorithms in the first part of this work, we demonstrate the implications of their usage in wildfire detection scenarios. To that end, several fire experiments under controlled conditions were conducted to study the behavior of thermal cameras in those situations to characterize how the raw sensor data are mapped to visually interpretable pseudocolor images. In this regard, attention is paid to the identification of the saturation levels of this type of sensor.

Third, we discuss the implications of the insights exposed and the potential developments in robotic perception towards autonomous UAV-based fire monitoring systems, namely through the identification of current roadblocks and possible enabling solutions that manufacturers should integrate for the widespread use of this technology.

After this introduction, the remainder of this article is structured as follows: Section 2 covers the overview of image processing algorithms employed in thermal cameras. Section 3 presents a data-driven method based on thermal imaging for fire situation awareness. Section 4 presents the experimental setups and conditions of the laboratory and field trials. Section 5 analyzes the results and discusses the implications for the integration of thermal imaging in robotic perception of wildfire surveillance. Finally, Section 6 presents the conclusions and offers suggestions for future research.

## 2. Thermal Imaging

The basic principle of thermal imaging is based on the concept of sensing the radiance emitted from objects of interest. Note that above absolute zero temperature, i.e., 0 K, all bodies emit thermal radiation. In a general definition, what is referred to as thermal range is comprised of radiation with wavelengths in the 10^−7^ to 10^−4^ m interval of the electromagnetic spectrum. Hence, this range includes spectral bands in the ultraviolet, visible, and infrared regions of the spectrum.

Although this range is rather ample, thermal cameras usually only cover a part of it, which varies depending on the model and the application for which it is intended. More specifically, the devices employed in this work are based on uncooled microbolometer detectors that cover part of the infrared band, namely the 7.5–13.5 $\times 10^{-6}$ m spectrum, which is widely common for camera models in the market.

**Figure 1:**
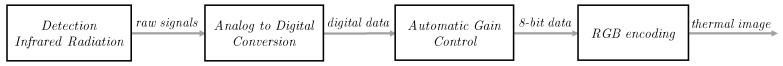
High-level diagram of the process flow in uncooled microbolometer arrays.

Thermal infrared cameras employ complex signal processing architectures in order to output images that convey relative temperature differences between objects in a scene [24]. This process comprises several stages, which are summarized in Figure 1 and are briefly described in the following.

In the first stage, incident infrared radiation is absorbed, inducing changes of resistance in each microbolometer detector of the focal plane array, which are translated into a time-multiplexed electrical signal by a readout integrated circuit (ROIC) [25]. Then, the array is calibrated automatically each time it is powered to match all microbolometers to the same input/output characteristic function that relates the measured radiance intensity and the output signal. This is performed through a linearization process and temperature compensation of the signals from individual detectors of the array [26]. Second, with these compensated signals, the measurements are transformed into raw pixel values that translate the intensity values that compose a monochromatic image. Raw image data can be subsequently transformed into pseudocolor images through an automatic gain control procedure and RGB encoding according to a user-specified color palette to facilitate interpretation.

In addition to sensing the radiation being emitted from objects in the field of view, another important aspect concerning thermal cameras is the ability to measure temperature. While this topic is extensively covered in the literature, with respect to how the incident radiation is transformed into approximate temperature readings [26], in this work, we do not delve into this matter for two main reasons. First, with this study, we aim to address the general image processing pipeline that is transversal to most thermal cameras, i.e., irrespective of these having radiometric capabilities or being non-radiometric. Second, although in different contexts, the correction of temperature values of thermographic images can be performed *a posteriori* using off-line post-processing methods, this requires a known reference in the image content [27]. In the case of wildfire surveillance, if we consider the environment to be open and unknown with regard to the temperature, i.e., without access to external absolute temperature readings, on-line thermal correction of the calibration for real-time applications is not possible.

Taking into account these considerations, the main focus of this work is on how raw digital readings are encoded into pseudocolor images, which can be applicable to image data from both radiometric and non-radiometric thermal cameras.

### 2.1. Sensor Characteristics: Preliminaries

Thermal imaging systems are optical instruments that are able to generate two-dimensional representations of the surrounding environment as a function of the strength of incoming thermal radiation. These exteroceptive sensors transform a digitally encoded array of pixels into an image according to the camera perspective projection, which depends on the focal length of the lens.

Digital images are generated according to the characteristics of the camera sensor, namely spatial resolution, temporal resolution and dynamic range [28]. The spatial resolution is intrinsically related to the size of the focal plane array and defines the number of pixels in an image, as well as the corresponding aspect ratio. The temporal resolution is associated with the operating frequency of the device, i.e., the frame rate at which the camera yields image data. In turn, the dynamic range corresponds to the interval of intensity values represented. Additionally, the resulting images also depend on the automatic gain, which is a fundamental aspect of the manner in which thermal images are encoded, since most camera models employ automatic gain control, as will be discussed further along.

To explore the effects of these characteristics, this work resorts to two distinct thermal cameras, namely the FLIR SC660 (FLIR Systems, Inc., Wilsonville, OR, USA) and FLIR Vue Pro (FLIR Systems, Inc., Wilsonville, OR, USA), which operate in the regions denominated as far infrared (FIR) or long-wave infrared (LWIR). The main specifications of these camera models are presented in Table 1.

Regarding the field of view (FOV) specifications and focal length, note that these cameras have distinct characteristics, which will influence the data recorded. Moreover, spatial resolution, temporal resolution and dynamic range also vary between both models, which is an aspect to take into consideration. In the case of the FLIR SC660, the automatic gain can be adjusted to different configurations to change the dynamic range.

In addition, keeping in mind the payload budget of small UAVs and comparing the weight and size of both cameras, only the FLIR Vue Pro has suitable characteristics to be taken onboard a small UAV. However, given the exploratory basis of this work, the radiometric capabilities of the FLIR SC660 are valuable for the study of these sensors in extreme conditions inherent to fire scenarios.

Although this work explores these cameras, other alternative models have been released more recently, with higher spatial resolution and gimbal integration, albeit at superior costs. Notwithstanding, from the standpoint of the hardware and firmware available, the update does not have a significant impact on thermal imaging results, since the major benefits of the newer models are the facilitated integration for deployment on UAVs or graphic user interfaces for control from mobile devices. Thus, the analysis presented herein also applies to different thermal cameras.

In the following, the image processing methods for generating thermal images are described through examples explaining the mapping algorithms that perform the transformation of raw digital data into pseudocolor-encoded images.

### 2.2. Mapping Raw Digital Data to Thermal Images

Thermal cameras are generally single-band in the sense that they only produce monochromatic images, which subsequently undergo a sequence of image processing steps to transform the raw digital data into pseudocolor images to highlight the details of the scene context. This processing pipeline can be implemented either on the device for storage or digital output, or on external software.

Currently, commercial off-the-shelf devices already provide a variety of color palettes to enhance the visual interpretation of the amounts of radiance captured by the sensors. However, to design intelligent algorithms for autonomous systems, the color-encoding schema have to be well suited for the robotic perception approach, which is essential for fulfilling the application requirements. For this reason, this also requires a deeper understanding of the image processing pipeline to leverage the potential of this type of sensor for novel applications.

The raw intensity levels are given by a digital number assigned by the sensor analog-to-digital converter, which can be of 14-bit or 16-bit order depending on the sensor bit resolution. In this work, we will employ both these alternatives, as specified in Table 1, but since these algorithms apply in the same way to both versions, the following examples showcase only the processing of raw data in 14-bit space. Further along in the analyses of the sensor response to fire scenarios both cases will be covered in detail. Note that the bit resolution relates to the temperature range of the camera, and for camera models with different modes, such as the FLIR SC660, the intensity level values will also be influenced by the camera configuration, i.e., the high-gain or low-gain modes.

To obtain a thermal image in pseudocolor, the image processing pipeline is divided into two main steps: (1) application of a data compression technique denoted as the automatic gain control; (2) application of the color palette specified, yielding images with three channels corresponding to the RGB color-space representation.

Automatic gain control (AGC) is a histogram-based technique that performs the transformation between raw data formats to 8-bit image data. This processing method is responsible for data compression, which implies a considerable loss of information. For the 16-bit case, from a range of possible values from 0 to 65,535, the resulting image will be represented with values in the 0 to 255 interval. To counteract the decrease in detail, the AGC algorithms are designed to enhance the image contrast and brightness in order to highlight the scene context.

The following sections cover with illustrative examples the main variants of AGC algorithms implemented in thermal cameras that practitioners should be aware of to leverage this technology. Then, the color-mapping schema and several available color palettes are presented in Section 2.2.3.

#### 2.2.1. Histogram-Based Automatic Gain Control

AGC methods are typically variants of the histogram-based operations widely used in computer vision for contrast enhancement, e.g., histogram equalization [28]. However, in thermal imaging, AGC also implies data compression between raw formats (e.g., 14-bit or 16-bit) into display-ready data (8-bit).

**Figure 2:**
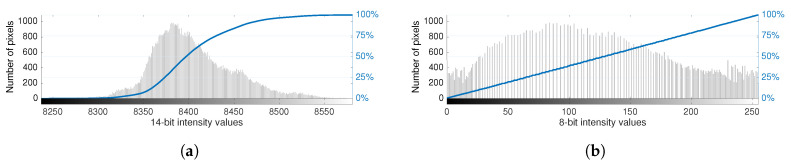
Automatic gain control with histogram equalization: (**a**) the raw data histogram is used to compute the cumulative distribution function (in blue) for the bounds of the 14-bit intensity values; (**b**) the data are compressed to 8-bit by flattening the distribution of the compressed data histogram, resulting in an approximate linear cumulative distribution function (in blue).

In classical histogram equalization, the nonlinear mapping used for contrast enhancement is derived directly from the cumulative distribution function (*cdf*) of the raw intensity values. This approach allows one to achieve an approximate linear *cdf* on the compressed 8-bit data, yielding an image with intensity values spread across the full extent of the available 8-bit range. Figure 2 illustrates this AGC procedure for a raw 14-bit image converted into the 8-bit range.

Note that although the bit resolution of the 14-bit sensor represents values up to 16,383, for environments with ambient temperatures around 20 °C, the raw data captured are represented in a narrow band of the full range, as can be observed in Figure 2. Therefore, compression and contrast enhancement play a pivotal role in the encoding of thermal images. However, note also that enhancement operations in thermal images artificially distort the data, meaning that the physical correlation that relates the radiant flux from infrared radiation and pixel intensity is lost.

**Figure 3:**
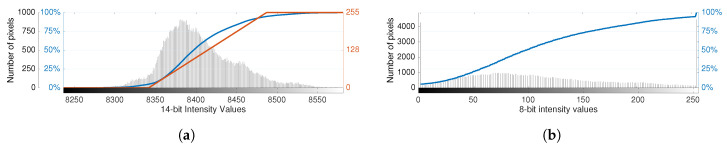
Automatic gain control with the linear algorithm: (**a**) the raw data histogram is used to compute the cumulative distribution function (in blue), for which a percentage of the values are clipped at the lower and upper bounds to obtain the resulting nonlinear transformation (in red); (**b**) the data are compressed through the previous transformation, resulting in the histogram for 8-bit intensity values with the corresponding cumulative distribution function (in blue).

Alternatively, for cases where it is important to preserve the correspondence between the pixel intensity and temperature of objects, for instance, a “linear” mapping function is better suited. The linear approach also relies on the *cdf* to define the image transformation table (ITT) by defining the slope and clipping points of the resulting nonlinear mapping function. Figure 3 depicts the application of this AGC algorithm to the previous example.

In addition to the *cdf*, the linear transformation, given by T(x)=mx+b, requires setting the midpoint of the image transfer table, ${\mathrm{ITT}}_{\mathrm{mid}}$, which is normally the average value of the range of the 8-bit range (128), and the tail rejection percentage $r_{\%}$. These parameters are used to determine the points on the *cdf* employed to define the linear equation and respective slope, *m*, and intercept, *b*, as:

(1) $$\begin{array}{cc} b & ={\mathrm{ITT}}_{\mathrm{mid}}-\mathrm{mean}\left(x_{100-r_{\%}},x_{r_{\%}}\right)\cdot m \end{array}$$

(2) $$\begin{array}{cc} m & =255/(x_{100-r_{\%}}-x_{r_{\%}}), \end{array}$$

with *x* representing the raw values indexed in the x-axis of the histogram. The image transformation function, $T^{\prime }\left(x\right)$, is defined for the range of values of the histogram by clipping the values to the lower and upper bounds of the output domain [0,255], as can be observed in Figure 3. Note that the clipping operation increases the absolute frequency of 0 and 255 intensities in the 8-bit histogram. Subsequently, the data are converted through the ITT look-up table.

In practice, the ITT midpoint influences the brightness of the image in the sense that increasing or lowering the midpoint shifts the equation horizontally to the left or right, respectively. As a result, this clips the data to zero at a corresponding lower or higher raw value, and vice-versa for the 255 upper bound. This aspect is especially relevant because, for fire detection scenarios that present high temperature gradients, both low and high raw values are important for situation awareness. Likewise, the definition of the tail rejection percentage follows the same principle. Therefore, parameter tuning should be approached with caution so as not to discard relevant data.

Nonetheless, the linear algorithm is not the most used AGC method because this compression technique implies a considerable loss of detail. To avoid this limitation, thermal cameras usually employ the plateau equalization algorithm, which aims to balance the distribution of all intensity levels in the image scene, thereby enhancing image contrast and highlighting differences in temperature.

The plateau equalization algorithm [29] implements a nonlinear mapping that compromises between histogram projection and histogram equalization. The concept is to bound the representation of the different intensity levels to a defined threshold, termed as the plateau value, *P*, while limiting the slope of the transformation function through the maximum gain value, *G*, which is set to 1 by default. However, for images with low dynamic range, where a small interval of values has high bin counts, the plateau equalization algorithm may yield fewer intensity values than the 256 available in 8-bit space. To ensure that the entire contrast depth is leveraged, the maximum gain sets the upper limit of gain that can be used to stretch the data to the full extent of the 8-bit range. Considering that fire detection applications most likely exhibit high dynamic range situations, this parameter will not be tuned in the development of this work.

**Figure 4:**
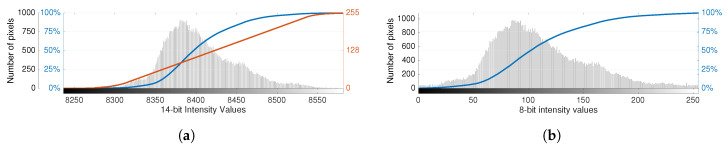
Automatic gain control with the plateau equalization algorithm: (**a**) the raw data histogram, with the respective cumulative distribution function (in blue), is clipped according to a prescribed plateau value to generate the transformation function (in red); (**b**) the data are compressed through the previous transformation, resulting in the histogram for 8-bit intensity values with the corresponding cumulative distribution function (in blue).

First, the image histogram is clipped according to the plateau value and represented through the effective count, $c_{x}$, for each raw intensity level *x*, which conditions the absolute frequencies not to exceed the prescribed plateau value, *P*, as:

(3) $$c_{x}\equiv \min\left(C_{x},P\right),$$

where $C_{x}$ represents the original count of pixels with raw intensity value *x*. The plateau equalization mapping function is based on the cumulative distribution function of the clipped histogram values, denoted by *cdf’*, which is computed according to:

(4) $${\mathrm{cdf}}^{\prime }\left(x\right)=\sum_{j=0}^{x}c_{x},\;0\leq k<2^{N},$$

with *N* representing the exponent corresponding to the original bit resolution of the sensor. Then, the transformation function based on plateau equalization for an 8-bit compression is defined as:

(5) $$T_{PE}\left(x\right)=\left\lfloor \frac{255\cdot {\mathrm{cdf}}^{\prime }\left(x\right)}{{\mathrm{cdf}}^{\prime }\left(2^{N}\right)}\right\rfloor ,$$

where ⌊⌋ represents the truncation operator for the next lower integer. Note that if the plateau value equals the maximum absolute frequency in the original histogram, this algorithm is equivalent to histogram equalization. In turn, if the threshold is defined as 1, this algorithm behaves as histogram projection. The plateau value is established as a percentage of the maximum value of the bin count of the histogram by default, e.g., 7%, but varies depending on the camera model and specifications. Figure 4 illustrates the application of the plateau equalization algorithm for the previous example.

Besides these algorithms, some cameras also have the information-based equalization variant that combines the plateau equalization algorithm with basic image enhancement techniques to yield more detail from scene context, irrespective of the data distribution. This method is not implemented in FLIR ResearchIR but was implemented herein for illustration purposes.

Information-based equalization aims to allocate a proportional amount of the dynamic range to different parts of the scene to capture the most information from the scene context, irrespective of these being represented with a large part of the image, such as the background, or by small areas with slight variations in temperature in the foreground. This method performs this by using a high-pass filter to highlight detail in the image, which is subtracted from the original image before the application of plateau equalization. The low-pass histogram is subsequently modified by increasing the bin count of the pixels in the high-pass image, hence increasing image contrast whilst also including greater detail. For instance, in a fire scenario, this may be useful to distinguish the fire while also being able to discern if there are people in the scene. Since this is an extreme case, with high dynamic range in the raw data, the effect of the compression into 8-bit data implies a significant loss of detail, therefore making more important these image enhancement techniques to highlight even subtle variations in temperature.

While in controlled environments, adequate tuning of this type of algorithm can yield better results, in outdoor contexts, adjusting these methods for a robust and consistent performance becomes very complex because the uncertainty and measurement errors associated with these open and unknown environments are greater due to the distinct emissivity properties of the multitude of heterogeneous materials that can be encountered [26]. In this way, by distorting the actual measurements, these contrast enhancement techniques also introduce greater inaccuracy in the image representation, which can hinder the development of robust robotic perception methods.

#### 2.2.2. Thermal Imaging Metadata

In addition to the image data, thermal imaging files also store different types of metadata encoded under standard metadata formats, e.g., TIFF, Exif and XMP. By accessing this information through adequate interfaces, several valuable parameters can be retrieved. In addition to encoding the standard properties of the digital camera (e.g., focal length and focal plane resolution) and GPS coordinates, the manufacturers can also store camera calibration parameters relevant for conversion between raw intensity values and temperature values.

Herein, we explore relevant metadata tags that help to shed light on the working mechanisms of these sensors, providing important insight into useful parameters for robotic integration. More specifically, since we tackle the relation between raw data and RGB-encoded data, only the parameters that influence this transformation will be addressed.

**Figure 5:**
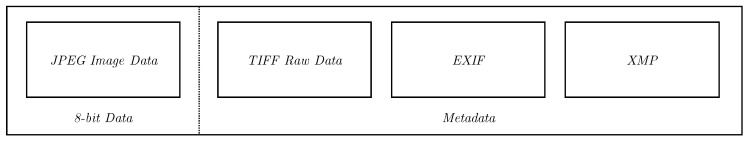
Schematic of file structure of FLIR image format.

The metadata were retrieved from raw video files and image files encoded in “SEQ” or “TIFF” and a proprietary format from FLIR that encodes both JPEG and metadata, as outlined in Figure 5.

By analyzing the metadata of the image and video files, two Exif tags were identified that are closely related to the histogram-based algorithms presented in Section 2.2.1, namely the raw value average and raw value range. The range of raw values and the average values are encoded separately in the metadata, but may not correspond to the values that would be computed from the raw frames for every instance. The reason for this is probably the proprietary in-camera processing to deal with noise and other types of outliers. For the FLIR SC660, the calculated values do not match the encoded values in the files, whereas for the FLIR Vue Pro, both match exactly for data recorded in image mode. This indicates that the update of these values also varies depending on the device and the mode of capture, as it is related to the firmware. With the average and the range of raw values from the metadata tags, the maximum and minimum values of the color scale are computed as follows:

(6) $$\begin{array}{c} \mathrm{Raw}\;\mathrm{Value}\;\mathrm{Max}=\mathrm{Raw}\;\mathrm{Value}\;\mathrm{Average}+\frac{\mathrm{Raw}\;\mathrm{Value}\;\mathrm{Range}}{2} \end{array}$$

(7) $$\begin{array}{c} \mathrm{Raw}\;\mathrm{Value}\;\mathrm{Min}=\mathrm{Raw}\;\mathrm{Value}\;\mathrm{Average}-\frac{\mathrm{Raw}\;\mathrm{Value}\;\mathrm{Range}}{2}. \end{array}$$

However, note that if these metadata tags are not available, the maximum and minimum values of the raw data can be computed directly on-line, as observed in the histogram-based algorithms presented in Section 2.2.1, by extracting maximum and minimum values directly from the raw frames.

Given that the histogram-based mapping functions and the respective color encoding adapt according to the scene context, these parameters are essential for understanding how the color scale adjusts over time. Thus, these will be explored next in the data-driven analysis approach proposed in Section 3.

Considering that fire surveillance applications are an extreme case with a high dynamic range, it is important to evaluate how these techniques behave in such scenarios. Thus, to delve into this issue, in the following, the color encoding used to enhance the interpretation of thermal data is presented, along with the comparison of an example for a controlled burn performed in a real-world context.

#### 2.2.3. Color Mapping

Following the conversion into an 8-bit image format, the data are represented in the 0 to 255 range. To encode these values in an RGB color space representation, the cameras offer several color palettes with distinct characteristics, which are adequate for different applications. In cases where the cameras provide the raw data in addition to the RGB-encoded images, these data can be post-processed with different color palettes for further analysis.

**Figure 6:**
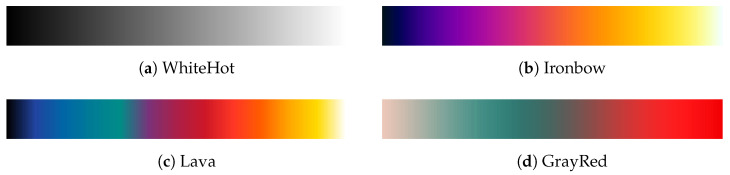
Examples of thermal imaging color palettes.

The color palette is a discrete set of color samples composed of values for each color channel, and it is defined in a look-up table (LUT) in the camera firmware or external software. In practice, this discrete sequence of values provides a continuous representation of the mapping of values in the image. Figure 6 illustrates this for some widely available color palettes, presenting the set of discrete colors that form the color mapping applied to the 8-bit data. The color sequences are depicted according to an increasing order of bit values, meaning that these are attributed from lower values to higher values of sensed radiant flux. Notwithstanding, recall that the color scale is adaptive; thus, the color assignment also depends on the AGC algorithm. For this reason, the full spectrum of colors is not necessarily used in each image.

The different types of colormaps depicted can be advantageous in different applications. For instance, WhiteHot or Ironbow employ a sequential colormap with a uniform distribution between two main colors. In turn, Lava uses the contrast between several colors to enhance subtle differences between temperatures of objects in the scene, whereas the GrayRed alternative employs a divergent color distribution to highlight large temperature gradients in a scene.

Since the principal interest herein is to adapt thermal imaging sensors for wildfire detection and monitoring, the GrayRed color palette was selected. As depicted in Figure 6d, this palette applies high-contrast colors with a divergent color scheme, which is useful to draw attention to the hottest objects in the scene. Furthermore, an extensive color-based data analysis also employing this colormap in the detection of fire situations was presented in previous work [22]. Thus, in the interest of extending the scope of this investigation to the analysis of both color-encoded images and the raw data, the same color palette will be used in this article.

**Figure 7:**
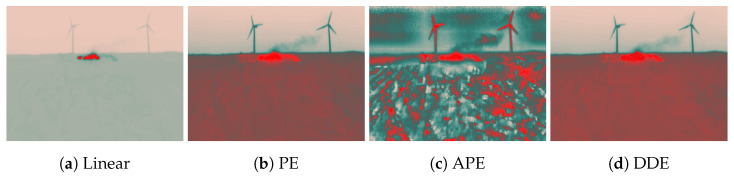
Comparison of results of automatic gain control (AGC) algorithms: (**a**) linear, (**b**) plateau equalization (PE), (**c**) advanced plateau equalization (APE), and (**d**) digital detail enhancement (DDE).

For comparison of alternative automatic gain control techniques, Figure 7 demonstrates the resulting images obtained with the different histogram-based methods for a controlled burn in a real context. The linear and plateau equalization (PE) methods follow the principles explained previously, whereas two additional proprietary FLIR methods that are available through FLIR ResearchIR are also presented, namely digital detail enhancement (DDE) and advanced plateau equalization (APE).

From observing these examples, it becomes clear that advanced contrast enhancement techniques degrade the ability to distinguish the fire from the surroundings. Note that these algorithms were applied with the default configurations, i.e., in their less intense modes, so further tuning of the parameters would deteriorate the image interpretation even further.

In addition to these core AGC methods, the image processing pipeline can also include, for some cameras, an array of proprietary features, such as active contrast enhancement (ACE) and smart scene optimization (SSO). These algorithms also tune the image output, and their parameters can be adjusted manually using manufacturer software applications, but in this study, the default values were used.

## 3. Data-Driven Thermal Imaging Analysis

Since fires are sources of extreme heat with a strong emission of infrared radiation, thermal imaging sensors can potentially bring significant advantages for fire surveillance applications. However, to implement autonomous systems for early detection and monitoring, i.e., independent of human-in-the-loop approaches, the robotic perception algorithms have to be data-driven.

To provide a preliminary intuition on the behavior of this type of sensor in the advent of a fire ignition, this section starts by introducing an experimental example of a fire detection instance in a controlled environment with static image capture conditions. Subsequently, a feature engineering approach is designed for data-driven situational awareness in fire scenarios.

### 3.1. Thermal Imaging in Fire Scenarios

**Figure 8:**
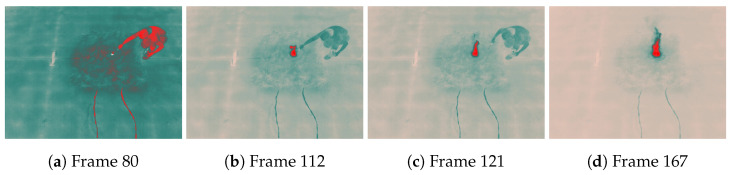
Fire ignition detection with a radiometric thermal camera.

**Figure 9:**
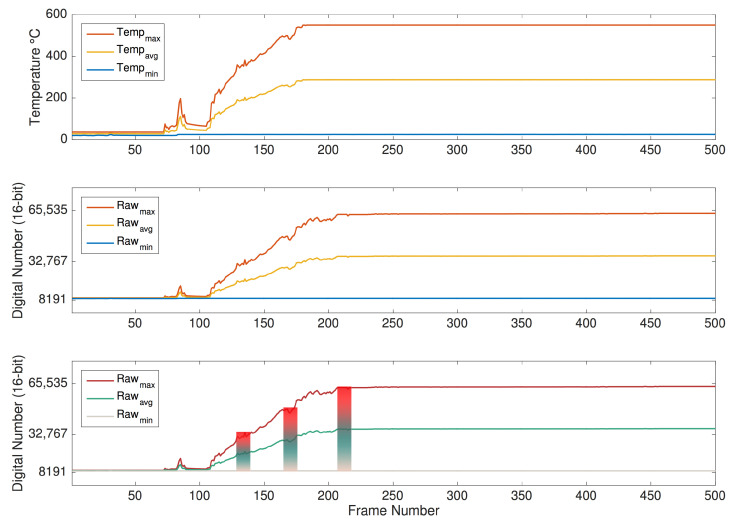
Comparison of temperature (**top**), raw data (**center**), and the evolution of the adaptive color scale (**bottom**).

The following example presents an illustrative demonstration relating image response, the temperature profile of the scene, and raw measurements to offer some insight into the sensor behavior. The experiment was performed in a laboratory environment with a direct line of sight between the fire and the thermal camera, in this case, the FLIR SC660. Figure 8 presents selected samples from the ignition of a straw fuel bed at different time instances. The corresponding temperature profile, which is represented in Figure 9, was extracted from the radiometric data provided through FLIR ResearchIR software. At this juncture, this example is presented as a preliminary analysis. For further information concerning the experimental setup, refer to Section 4, where the experiments are covered in full detail.

Observing the samples depicted in Figure 8, it should be noted that although the strongest source of thermal radiation is depicted in red, this does not mean it is necessarily fire. Hence, it would be a naive approach to focus only on the detection of the hottest objects in the scene. This is caused by the adaptive nature of the histogram-based algorithms in the image processing pipeline.

In the first frame, right before the ignition, the majority of the frame has green-colored pixels, whereas from the second frame onwards, as the fire starts, the contrast progressively shifts. As a result, the objects with temperatures close to the ambient temperature are subsequently represented by pixels with a whiter shade. It is important to notice that, in this context, the color scale that translates the differences in temperature adjusts with respect to the object in the scene with the maximum temperature. This effect can be observed by comparing the first and second frames. Whereas in the first, the person can be observed in red, in the second, the person is depicted in dark green. This happens because radiation being emitted by the greatest heat source is measured by the sensor with a much higher intensity than the radiation emitted by the person. While the temperature of the person remained unaltered, the range of raw measurements expanded, prompting a significant adjustment in the color scale. The underlying reason for the color evolution is that, at this stage, the body temperature is much closer to the ambient temperature than to the temperature of the fire. Comparing the first and last frames highlights that as the temperature differences increase, the contrast in the image adjusts accordingly.

The temperature profile of the experiment is illustrated in Figure 9, along with the raw statistics computed from the data frames. Note that the temperature profile depicts the maximum and minimum values of temperature extracted from the pixels in the image, as well as the average temperature computed with the maximum and minimum values. Note that the first increase in maximum temperature around frame 80 concerns an ignition that did not fully develop, so a re-ignition was promptly conducted, which can be observed by the increase in maximum temperature from around frame 110 onwards.

In juxtaposition, Figure 9 also represents the corresponding evolution of the raw values, including the maximum and minimum raw values extracted from the image, as well as the average between these two metrics, to illustrate how the color scale adjusts as the dynamic range extends. Note that this depiction does not take into account the application of histogram-based algorithms, in the sense that it only translates a linear application of the GrayRed colormap to the raw values. Nevertheless, it provides a broad intuition regarding the adaptive color scale while relating the raw measurements and the temperature data with the pseudocolor images presented in Figure 8.

Since the camera was configured to measure up to 500 °C for this trial, it is noticeable by observing both graphs that the sensor saturates when that temperature is reached.

The analysis of these variables highlights the importance of testing the thermal imaging cameras in real operation scenarios and characterizing the behavior of these sensors in extreme conditions, as they may exhibit differences depending on the camera model. In the case of the FLIR Vue Pro in image mode, the values in the metadata match the maximum and minimum values of the raw frames.

Therefore, in order to employ thermal imaging sensors for situation awareness in fire detection and monitoring scenarios, it is important to translate the insights from this analysis into meaningful features for robotic perception, as will be explored in the following.

### 3.2. Feature Engineering

Autonomous robotics require real-time processing of sensor data for navigation and guidance, e.g., to enable sensor-driven planning, as it is crucial for the robot to have precise estimates of its position and attitude, as well as its surroundings, in order to plan its trajectory on-line. Even though emerging on-board computing platforms have higher processing capabilities, to incorporate powerful exteroceptive sensors capable of generating high-resolution samples of the surrounding environment, it is essential to handle the burden of data dimensionality. Furthermore, considering the required high sampling frequencies suitable for these tasks, mobile robotics applications are increasingly data-intensive; thus, feature engineering plays a prominent role in deriving efficient algorithms.

In light of the insights exposed in the previous sections, the following presents a feature engineering approach for situation awareness using thermal imaging, which combines feature extraction from raw data and pseudocolor images to yield relevant features for robotic perception in fire scenarios.

#### 3.2.1. Raw-Based Features

The feature extraction approach for raw data is based on the construction of features that aim to preserve the intuition of the actual working mechanism of thermal imaging sensors and the histogram algorithms of the image processing pipeline. Recalling the examples presented in Section 2.2.1, the raw-based features will explore the maximum, minimum and average values of the range of the raw data frames. As explained in the metadata description in Section 2.2.2, these variables can be computed according to Equations (6) and (7), but since these do not always match the metadata tags for the cameras tested, it is advisable to extract these variables on-line from the raw data frames.

#### 3.2.2. Color-Based Features

**Figure 10:**
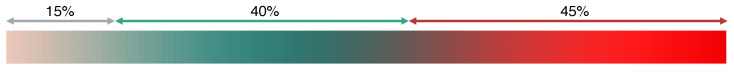
Division of the color scale [22].

To extract features that translate the evolution of the color statistics of the image in fire scenarios, as observed in Figure 8, this work employs the color segmentation heuristic proposed in [22]. The approach divides the GrayRed color palette into three parts according to the three main colors that compose this palette: gray, green and red. The complete color scale that comprises 120 distinct color representations defined in the RGB color space is partitioned with the division illustrated in Figure 10.

The first segment, represented in gray, corresponds to lower raw values, i.e., lower temperatures, and is defined by 18 color levels, which represent 15% of the color scale. The green segment is defined by 48 color levels, which represent the mid-range of raw values, corresponding to 40% of the color palette. Since this color palette is designed to draw attention to the hottest elements in a scene, the largest portion, corresponding to 45% of the color scale, is dedicated to 54 red color levels. Although these colors exhibit high contrast between them, color gradients between each color are low. The segmentation is performed according to the parameters in RGB color space defined in Table 2.

In contrast with strictly data-based approaches, the proposed feature engineering method allows the incorporation of expert knowledge about the sensor by deriving features from the information retrieved from raw data in combination with the data-driven color segmentation heuristic that describes the behavior of the sensor over time. Furthermore, these features are designed with data interpretability in mind, which is especially important for safety-critical applications, such as wildfire detection and monitoring systems.

In order to study the response of thermal imaging sensors, a comprehensive set of experiments was conducted for analysis of the sensor image processing pipeline and its application in real scenarios, which are presented in the following.

## 4. Controlled Fire Experiments

This section presents a set of controlled fire experiments performed in laboratory and field contexts. The trials comprise the burning of wild fuels, such as straw, common heather (*Calluna vulgaris*) or Baccharis (*Baccharis trimera*), and artificial materials present in a caravan that was also experimentally burned. In the following, each section describes the experimental setup and conditions used in the trials, as well as the means employed for data acquisition.

### 4.1. Laboratory Test

The laboratory experiment enables testing of the response of the camera to a fire ignition in a controlled environment, allowing a high-resolution sampling of the phenomena under static environmental conditions. Since the test was performed indoors, there was no influence of wind. The horizontal fuel bed was composed of straw with a fuel load of 600 g/m^2^ and a moisture content of 13%. The environmental temperature and relative humidity were 20 °C and 78%, respectively. By using these testing conditions, the behavior of the sensor and the subsequent image processing is not affected by external factors.

**Figure 11:**
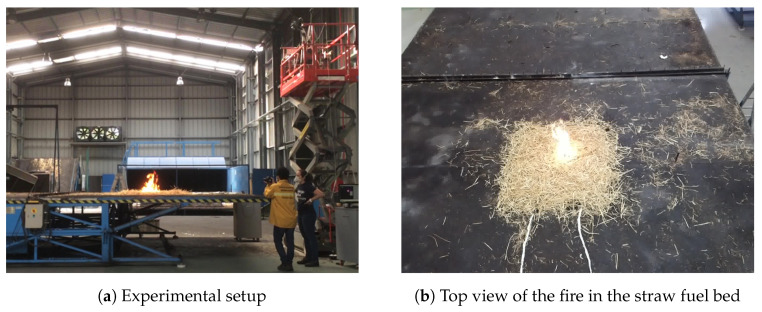
Laboratory trials with the FLIR SC660 mounted on an elevated platform.

For data acquisition purposes, the FLIR SC660 was positioned on top of an elevated platform with a direct line of sight to the fire, as depicted in Figure 11. The line of sight made an angle of 45° with the horizontal plane. In this setup, the data were processed and recorded in real-time using FLIR ResearchIR software through a cable connection to a desktop computer.

The objective of this type of test centers on capturing the transition to a fire scenario; thus, it requires a high frame rate. In this case, the device was configured to a 15 Hz frame rate.

### 4.2. Caravan Burning Test

The caravan burning test comprised the burning of man-made fuels, unlike the other trials, which only included natural fuels. This scenario is important to take into account, especially for airborne fire surveillance in wildland-urban-interface regions. In addition, due to the presence of some materials like plastic, the temperatures expected are much higher than those registered for wild fuel burning. The test was performed with average values of relative humidity of 75% and temperature of 23 °C. The wind velocity was below 2 m/s.

**Figure 12:**
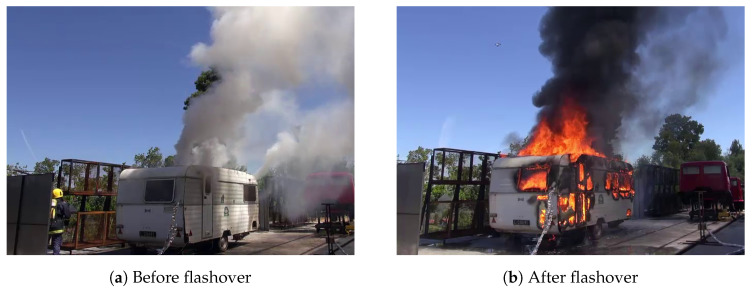
Recording of the caravan burning trial.

Like in the previous trial, the device used for data acquisition was the FLIR SC660 operated in connection with FLIR ResearchIR software with a 15 Hz frame rate. In this case, the camera was mounted on a tripod at ground level at approximately 10 m from the caravan, which is depicted in Figure 12.

To study this case, as fire-related temperatures reached higher levels, the automatic gain control configuration was changed manually mid-trial to expand the measurement range to cover higher temperatures.

In this trial, unlike in the previous example, there was no direct line of sight to the fire at the point of ignition, which occurred in the interior of the caravan, as can be observed in the visible range image presented in Figure 12a. Indeed, only after the flashover, i.e., the point where the heat and pressure build-up reached the maximum immediately before breaking the caravan wall, the fire started to be visible in the visible range.

### 4.3. Summer Festival Trials

To design algorithms with robustness in real-world contexts, it is crucial to analyze data acquired in highly dynamic scenarios. In that sense, a set of tests was performed at the venue of a festival outdoors in midsummer to emulate conditions of real operation of fire safety systems. The trials were conducted in a region with dry weather in the summer, with strong winds and high temperatures, when conditions of sustainable ignition propensity were consequently high as well. More specifically, during the period of the trials, the following meteorological conditions were registered: minimum humidity of 17%, maximum wind speed of 21 km/h, and temperatures up to 36 °C.

**Figure 13:**
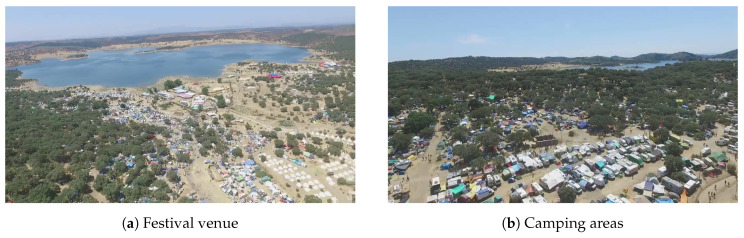
Festival venue and camping areas during the event where the trials were conducted.

The tests performed encompassed the aerial surveying of the festival area at several different locations. Figure 13 illustrates part of the venue surroundings, and provides some detail on the nature of the camping areas. As can be observed, the festival occurred in the fire-prone area, where the vegetation of trees and shrubs is very combustible. In addition to the vegetation, the inclusion of more combustible materials, such as tents and caravans, increases fire risk, which is exacerbated by the human presence, raising fire safety concerns.

**Figure 14:**
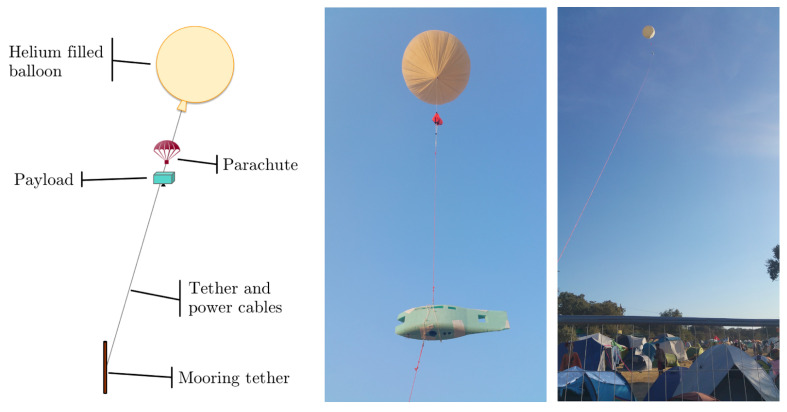
Thermal camera setup on tethered helium balloon.

To provide aerial monitoring for several hours to detect possible fire ignitions, the thermal camera was installed on a tethered helium balloon, as depicted in Figure 14. With this setup, the experiments were conducted at various locations of the venue to test distinct conditions. There were no fire events in these field tests, so for the purposes of this study, a fire in the rocket stoves of the community kitchens was used instead.

Concerning image acquisition, due to the limited payload budget of the balloon, only the FLIR Vue Pro has suitable weight to be installed onboard. The camera was programmed to capture images with a 5 s time-lapse, recording both the raw data and the pseudocolor images encoded with the GrayRed color palette in the camera storage.

In contrast to previous trials, where the acquisition conditions were static, since these experiments employed an aerial platform without active means of actuation for stabilization, the dynamics of the balloon influenced the image acquisition by varying the image field of view as a result of wind disturbances.

### 4.4. Mountain Range Field Trials

In addition to the previous experiments, several tests were performed in a real-world scenario in mountain range field trials to test the capabilities of the sensors in a long-distance monitoring scenario. For these tests, the main interest was to evaluate the performance of a compact thermal camera, namely the FLIR Vue Pro, for future airborne monitoring operations. The trials were undertaken at an altitude of over 1000 m with the following meteorological conditions: wind speed in a range of 13–16 km/h from the northwest, average relative humidity of 45%, and average temperature of 21 °C.

**Figure 15:**
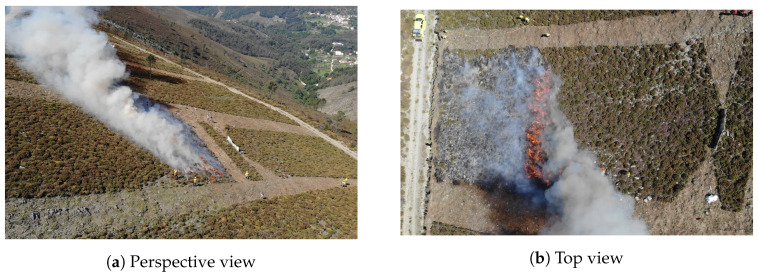
Experiments in mountain range field trials.

Figure 15 illustrates the experimental conditions of the trials. The field experimental burns were conducted on a hill with an average slope of 25%, and comprised the burning of shrubland vegetation, including common heather (*Calluna vulgaris*) and Baccharis (*Baccharis trimera*).

Concerning the image acquisition, in the trials, both thermal cameras were employed, but given the aforementioned objective of these experiments in particular, this work only explores the data from the FLIR Vue Pro. The images were captured at ground level from across the valley at around the same altitude as the field burnings, with a direct line of sight between the camera and the fire at a distance of approximately 600 m. The data acquisition was configured to record the data in the camera storage, saving both the raw data and RGB-encoded images with the GrayRed color palette at a rate of one frame per second.

## 5. Results and Discussion

This section presents the results of the application of the data-driven thermal imaging analysis to the controlled fire experiments performed in laboratory and real contexts according to the specifications presented in Section 4. As previously introduced, the proposed approach explores raw-based features, as well as the color-based features derived from the segmentation heuristic proposed in Section 3.

### 5.1. Laboratory Test

In the laboratory trial, under static conditions of image acquisition, it was possible to record with FLIR SC660 the transition from a situation without a fire to a fire scenario through a high-rate sampling that includes the point of ignition. This procedure enables capturing the influence this phenomenon produces in the data acquired.

**Figure 16:**
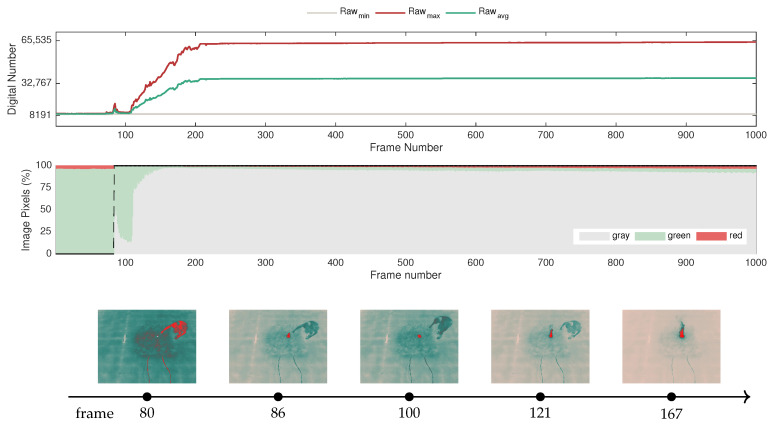
Feature-based representation of the sensor response of the thermal camera to a fire ignition: raw features (**top**); color features (**center**); sample frames (**bottom**).

Figure 16 depicts the evolution of the raw-based features, illustrating how the dynamic range expands as the fire develops. In turn, it also intuitively demonstrates how the variation of the dynamic range influences the color statistics of the images. The sample frames concern the transient stage immediately before and after the start of the fire. Note that the first small peak around frame 80 occurs in a first attempt at the ignition, but as the fire does not fully develop, the straw was reignited. The fast response of the sensor indicates that it is sensitive enough for this type of application.

In Figure 16, it is possible to identify that the sensor undergoes three distinct states: (i) before the point of ignition; (ii) transient state; (iii) steady-state. As indicated in the figure, at first, the environment is in equilibrium, with generally green levels corresponding to the mid-range. Note that the dynamic range in this state is narrow, since the incoming radiation originates predominantly from objects at ambient temperature.

In the second stage, which is prompted by the fire ignition, the camera detects different strengths of incoming radiation corresponding to a severe temperature gradient and, as a result, the dynamic range starts increasing. This drives the adaptation of the color representation according to the histogram-based algorithms presented in Section 2.2. Consequently, the increase in the percentage of gray pixels is prompted by the fire ignition, represented in red, since it is the hottest object in the scene and has a strong emission of infrared radiation, whereas objects at ambient temperature remain at the previous raw level.

In the third stage, a new equilibrium state emerges after the sensor stabilizes shortly after the beginning of the fire, at approximately frame 167. However, in these circumstances, due to the extended dynamic range, the color statistics have a considerably different representation with a high percentage of gray shades in the image context.

For this case, it is important to take into account that this experiment captures the image sequences with a frame rate of 15 Hz; thus, it is possible to record the transition between the equilibrium states. For cases with lower sampling frequency, the data may not capture this behavior, but only similar conditions resembling the first and third states.

Note also that although the dynamic range adapts over time, its measurement range is not altered; as such, the complete bit resolution is used in this configuration. Accordingly, since the measurement range is set between −40 and +550 °C, the raw measurements saturate at a digital number that would correspond to these temperatures; in this case, these match the full bit resolution from 0 to 65,535.

### 5.2. Caravan Burning Test

In the caravan burning test performed in an outdoor environment, as described in the experimental setup, the image acquisition conditions were also kept static, with the FLIR SC660 configured to capture image sequences at 15 frames per second. While this setup also allows for a high sampling of the phenomenon, in this case, the fire ignition is not in direct line of sight because it occurs inside the caravan. For this reason, it is also necessary to characterize how these circumstances impact the behavior of the thermal cameras in such scenarios. Moreover, the measurement range of the camera was adjusted mid-trial to encompass an extended range of temperatures. Consequently, this allows the evaluation of how the scaling of the bit resolution affects the raw digital data values.

**Figure 17:**
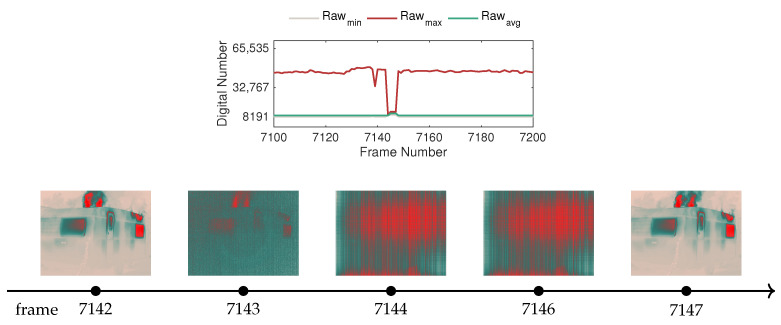
Effect of temperature-dependent non-uniformity correction (NUC), represented by selected samples of the raw data (**top**), along with the respective color-encoded frames (**bottom**).

However, before delving into the trial results, the effect of automatic temperature-dependent non-uniformity correction (NUC) should be addressed. This method consists of an internal process employed in thermal cameras to deal with spatial non-uniformity (NU) noise, which produces a fixed pattern over the thermal images that varies in intensity due to the internal temperature instability of such cameras [24]. To that end, on occasion, the camera performs a NUC reset, which results in a disturbance in the data acquisition process. Figure 17 exemplifies an instance illustrating this behavior along with relevant sample frames.

In the selected window of the raw-based features depicted in Figure 17, it can be observed that when the camera triggers the NUC reset in frame 7143, the raw values exhibit a sudden drop, which continues for several instants until frame 7146. Given that this behavior is sporadic, but it is expected to occur, as was verified for the case in the caravan burning test, appropriate filtering of this type of frame should be included in the image processing pipeline in automatic systems, particularly if integrated in autonomous navigation of robotic platforms for early detection and monitoring. In this work, a median filter was used with a window of 15 frames, which introduces a one-second delay in the case of the FLIR SC660, since this camera operates at 15 Hz.

**Figure 18:**
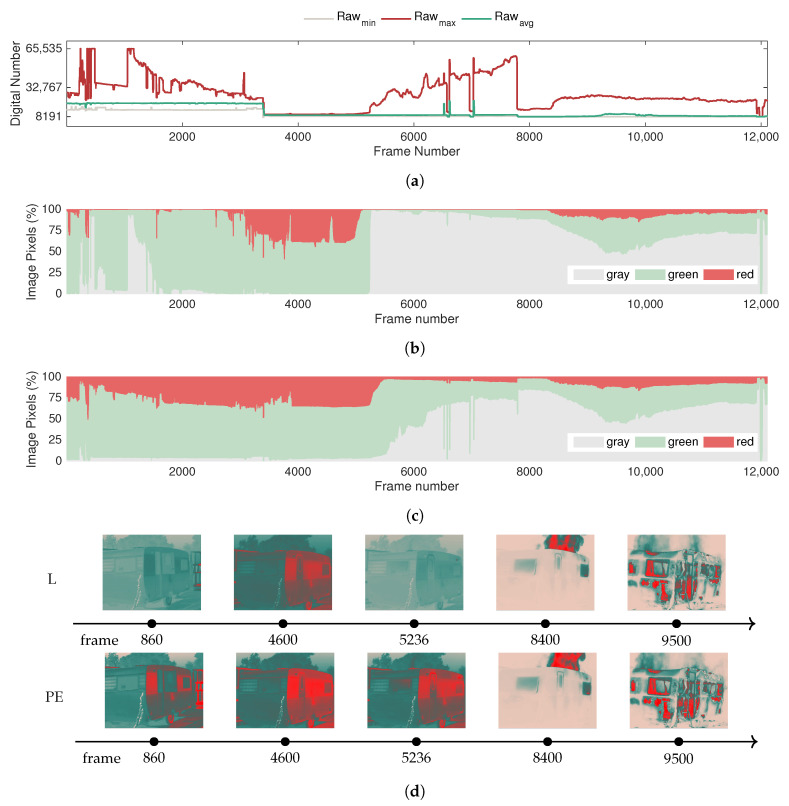
Representation of the filtered sensor response of the thermal camera to a fire ignition inside the caravan: (**a**) raw variables; (**b**) color-based features with the linear AGC (L); (**c**) color-based features with the plateau equalization AGC (PE); (**d**) comparison of frames between both approaches.

Figure 18 illustrates the variation of the features extracted from the raw data in comparison with the color features derived with the color segmentation heuristic for images encoded with a linear histogram approach (L) and the plateau equalization algorithm (PE) in the second and third graphs, respectively. The data presented in the graphs were pre-processed to filter out the effect of the NUC frames. In this example, we present the sensor response for two automatic gain control methods: linear (L) and the plateau equalization (PE).

Observing Figure 18, the adjustment of the measurement range from −40 to +550 °C to the upper temperature limit of +1500 °C that occurs at frame 3406 has a prominent impact on the raw data, which can be identified by the steep drop in the raw features at frame 3406. Notably, the scaling of the bit resolution amplifies the measurement range, but at first, this results in a narrow distribution of the raw features at lower bit values.

However, as the fire starts around frame 5100, the dynamic range begins to expand. With the evolution of the intensity of the fire, the dynamic range extends to comprehend higher values within the available resolution, reaching a maximum of 52,343 at frame 7736, corresponding to a maximum raw value of 60,978 and minimum raw value of 8635.

Subsequently, the sudden drop at frame 7782 indicates a second adjustment of the measurement range. The flashover point occurs at frame 8310, and as a result, the fire starts burning the exterior of the caravan with greater intensity, as can be seen in the corresponding frames (8400 and 9500). In addition, it can also be verified that with the fire visible in plain sight, the sensor response tends to stabilize.

Concerning the color-based features, the effect of the fire starts to be detected by the sensor around frame 5236, as indicated by the increase in the gray pixel percentage and as had been verified in the previous test. This occurs precisely when the fire burns a small hole in the upper right side of the exterior of the caravan, as depicted in frame 5236 (see the frame from the linear approach). In frame 8400, the effect after the flashover can be observed, where the fire can be seen in plain sight, causing the contrast in the scene to considerably increase. Even with mid-trial adjustments in the measurement range, the behavior of the color segmentation heuristic is generally stable.

Comparing the response in terms of color features for the linear (L) and plateau equalization (PE) algorithms, it becomes clear that the linear approach can detect the beginning of the fire in a prompt manner, as can be observed by the abrupt increase of the percentage of gray pixels. Conversely, for the plateau equalization algorithm, the response occurs more gradually, but it is still possible to detect the fire with this color segmentation heuristic. However, this difference in terms of image-based features is an important aspect to consider when developing intelligent fire detection systems to operate in real-time.

Following the analyses presented regarding tests performed in controlled environments, to validate the insights exposed, the next sections concern field trials undertaken in real contexts.

### 5.3. Summer Festival Trials

In the tests performed at the summer festival, the FLIR Vue Pro was installed onboard a tethered helium balloon, as illustrated previously in Figure 14. Although the movement of the balloon enabled surveying a wider area of the venue, for several periods of up to 2 h long, given that this unactuated aerial platform moves according to the direction of the wind, the image capture is considerably less stable since the field of view of the camera drifts due to wind disturbances. Due to payload restrictions, there was also no active means of camera stabilization; therefore, the sensor response for this set of data will inherently be more stochastic.

Notwithstanding, the proposed feature engineering approach is theoretically viable for images acquired from mobile platforms because it employs features extracted from each frame, but does not rely on temporal dependencies. Hence, the analysis of these experiments is paramount for validating the applicability of this technique for integration in robotic perception pipelines for mobile platforms in the context of wildfire surveillance tasks.

**Figure 19:**
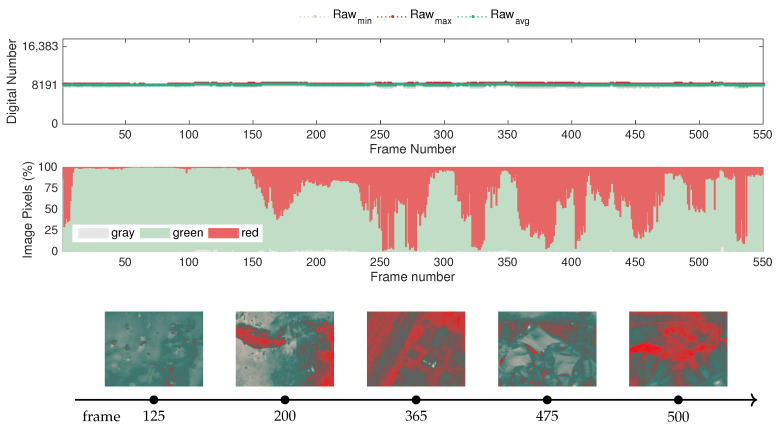
Results for data acquired when surveying the festival venue: raw features (**top**); color features (**center**); sample frames (**bottom**).

**Figure 20:**
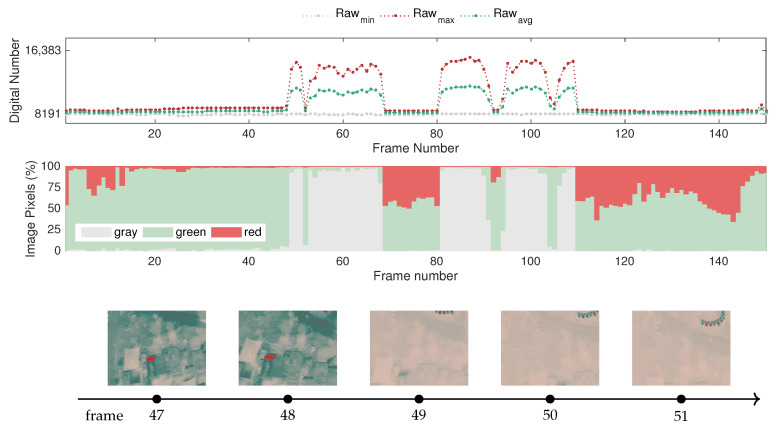
Results for data acquired when surveying the community kitchens area: raw features (**top**); color features (**center**); sample frames (**bottom**).

To that end, from several tests carried out in different parts of the festival venue, two examples were selected to showcase representative situations encountered in real contexts. The first example, presented in Figure 19, comprises a test in which fire situations were not detected, whereas the second case, depicted in Figure 20, covers areas without fire and also parts of the venue where fire was detected, namely in the community kitchens where rocket stoves were installed. The images were recorded with a 5 s time lapse between frames at an altitude from the ground of approximately 80 m.

Observing Figure 19 with respect to the evolution of the raw-based features, it becomes clear that under normal conditions, the sensor of the camera does not exhibit significant variations. Considering that the measurement range would allow the sensing of incoming infrared radiation up to a dynamic range of 16,383, i.e., the upper bound saturation level, the data demonstrate that in scenarios without fire, the maximum and minimum raw values do not exceed 8841 or go below 7812, respectively. Note also that in these trials, ambient temperatures reached up to 36 °C, so it is expected that the surfaces of objects captured in the field of view of the camera may have exceeded that value due to the different absorption properties of materials in such a heterogeneous context. Hence, including the raw-based features in detection and monitoring algorithms is rather promising for avoiding false alarms.

Conversely, since the festival took place in the summer, it is also normal to capture objects for cooling purposes that are at temperatures below the average ambient temperature. While this does not seem to have a considerable effect in the magnitude of the raw values, this effect can be observed according to the representation of the color-based features over time. More specifically, as different areas of the venue were surveyed, the color statistics vary considerably, predominantly influencing the red and green pixel percentages. For instance, in frame 365, it is possible to observe that a small set of items at cool temperatures caused the adaptation of the color representation, making the surroundings become depicted in red, even though there was not a sudden increase in temperature. In this way, since the histogram-based algorithms promote highly nonlinear effects in image data, color-based features require careful interpretation.

In turn, the presented sample frames also demonstrate that the appearance of red hotspots can be illusive, as it does not necessarily mean that fire sources exist in the scene, but rather that these areas are slightly above the average temperature. The representation of the color features takes this into account, since it considers in the color segmentation that the upper 45% of the scale is represented by red-shaded tones, as illustrated in Figure 10. The uneven distribution of colors in the GrayRed color palette also justifies the behavior of the sensor mentioned in the previous comment, particularly regarding how the presence of cool objects in the scene easily makes red the most dominant color in the image.

Concerning the second case represented in Figure 20, which includes situations at the venue where fire was detected, the behavior of the sensor was consistent with that in the controlled fire scenarios discussed previously. In normal situations, the raw features followed the same behavior of the baseline case presented in Figure 19, but when covering the community kitchens area, the dynamic range expanded due to the presence of fire sources. More specifically, regarding the complete sequence, the maximum raw value reached 12,477, the minimum raw value was 7918, and the maximum amplitude of the dynamic range, recorded in a fire detection instance, was 7290, which is well above the variations registered in the baseline case.

Accordingly, the color representation also suffers an abrupt adjustment, with the surroundings becoming depicted mostly by gray pixels. This effect can be attested in the accompanying sample frames illustrating the first detection instance, which were captured while covering the community kitchens area. Since fire was strictly forbidden in the festival premises due to fire safety concerns, with the exception of specific fires lit and controlled by the organization for scenic purposes and the community kitchens area, the remaining detection points in this sequence also concern the same area illustrated in Figure 20.

Considering that in these field trials, the images were captured from a considerably longer distance than in the laboratory tests, the data demonstrate that the color scale adapts even when hot objects have low spatial resolutions, which is essential for allowing early fire detection in aerial surveillance scenarios, therefore confirming the validity of the proposed approach for fire detection using mobile platforms.

Furthermore, recalling that the FLIR Vue Pro has a more limited measurement range, with an upper saturation level at 150 °C, this may raise some concerns regarding false alarms. However, under real operation conditions, no false detections were encountered.

### 5.4. Mountain Range Field Trials

In the field trials conducted in a mountain range, the images were captured from a longer distance than in the previous tests. The purpose of these tests was to evaluate a key issue regarding the use of compact thermal cameras, particularly concerning their capabilities when operating outside the nominal conditions specified in the official hardware datasheets. The field trials performed allow the evaluation of the behavior of the camera in a long-distance image capture setup to assess if the signal-to-noise ratio in this scenario is sufficient for fire detection applications. In this case, the tests were performed with the FLIR Vue Pro, which was configured to record images with a 1 s time-lapse at a distance of approximately 600 m, as specified in the description of the experimental setup (Section 4.4).

**Figure 21:**
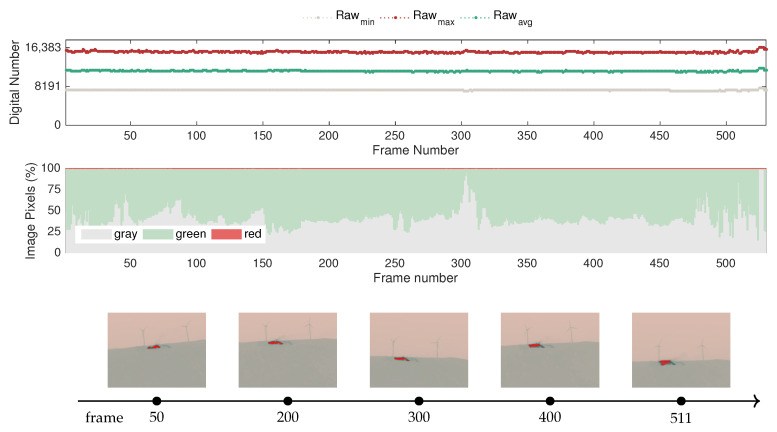
Results for fire detection at long distance in a mountain range: raw features (**top**); color features (**center**); sample frames (**bottom**).

**Figure 22:**
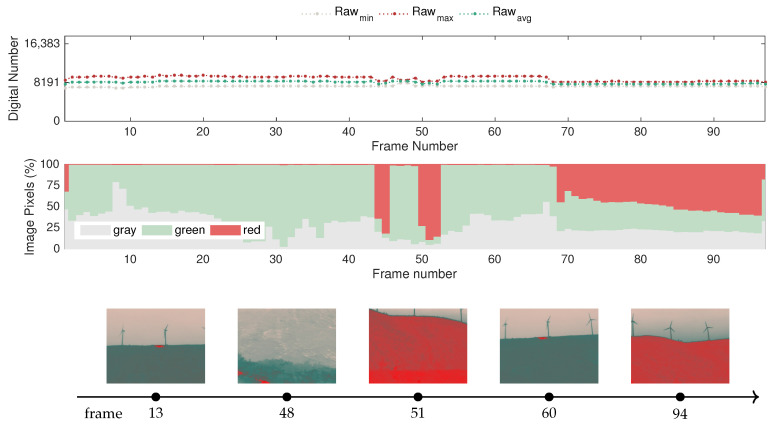
Results for the fire aftermath captured at long distance in a mountain range: raw features (**top**); color features (**center**); sample frames (**bottom**).

From the several tests performed in the mountain range, two cases were selected for analysis, encompassing a situation with an active fire, which is presented in Figure 21, and another in the aftermath of a fire, depicted in Figure 22.

Regarding the monitoring of an active fire scenario, it can be observed in Figure 21 that the statistics of the raw variables do not exhibit particular variation, remaining with an extended dynamic range for the several minutes featured in this trial. Notably, despite the long distance, the sensed infrared radiation does not suggest a significant signal attenuation, as the sensor bit resolution is used to a great extent. Indeed, the maximum raw value registered is the sensor upper saturation level at 16,383, while the minimum raw value recorded is 7235, which corresponds to pixels representing the sky in this case, as can be confirmed in the sample frames.

For this case, the evolution of the color-based features does not reveal particular changes and is consistent with the insights exposed in the previous trials. The sensor response stays generally constant, with slight variations attributed to the change of field of view of the camera. The gray and green levels dominate the image context, and a reduced percentage of red pixels depict the area of the fire, as can be observed in the sequence of frames selected.

Although the FLIR Vue Pro was not specifically designed for this type of task, these results validate the capability of detecting fire sources at long distances. In turn, an important aspect to take into account when considering extended distances is that due to the limited resolution of the focal plane array and, consequently, the output image resolution, with the increase in distance, the minimum size of the fire that can be detected naturally increases as well.

Since wildfires often extend over large periods and can be inactive after firefighting operations take place, but re-ignite before the fire event is completely extinct, the monitoring of areas in the fire perimeter is important for preventing rekindles. In this context, the ground is usually at higher temperatures, adding difficulty in pinpointing potential re-ignition spots.

The following test, performed in the field trials and targeting the aftermath of a controlled fire, which is depicted in Figure 22, aims to uncover how these circumstances differ from the previously studied cases.

Observing the time-series of the raw-based features, it is immediately noticeable that the dynamic range is reduced once again, but with visible gaps between the minimum, average and maximum values, unlike in the baseline case encountered in the festival trials. In contrast, in this case, the maximum raw value extends as high as 9782, the minimum raw value remains around 8141, and when the dynamic range is greater at 2426, the average raw value is 8569.

Concerning the adaptation of the color-based features, it should be noted that the field of view of the camera was changed considerably to capture distinct parts of the monitoring area, as illustrated by the set of sample frames presented in Figure 22. In this case, the green and gray percentages are predominant when capturing the burned area, which is depicted in red in frames 13 and 60 because it is at a higher temperature than the surroundings. The sky area remains depicted in gray; however, in contrast with the situation with an active fire, the ground is represented by a considerably darker tone of green. In that sense, the color-based features alone do not evidence a difference from the previous case, which highlights that the color segmentation heuristic has to be fine-tuned depending on the image capture setup or defined with a more restrictive range for the green class. Interestingly, when the burned area is not in the field of view of the camera, the ground is generally depicted in red, as can be observed in frames 51 or 94.

In turn, when facing downward on the valley, as illustrated in frame 48, the distance influences the color representation, with closer objects represented by the bottom part of the image, and the gray part representing the mountain on the other side of the valley. This result shows that in addition to being capable of detecting fire at long distances, the thermal sensitivity of this sensor is also able to convey depth perception. While this does not play a significant role in the detection in itself, it has interesting implications for autonomous navigation by robotic platforms.

### 5.5. Discussion

From the analyses presented throughout this study, it becomes evident that thermal cameras can potentially bring improved reliability for fire detection and monitoring systems in several aspects in which visible range systems have limitations. On the one hand, the proposed data-driven feature engineering approach that combines expert knowledge on the sensor working principles with color-based features derived from processed image data was demonstrated to be robust to false alarms, even when tested in highly dynamic environments. On the other hand, it was possible to validate the applicability of commercial off-the-shelf camera models in long-distance scenarios for fire detection applications, as verified with the onboard compact thermal camera.

However, adapting thermal imaging cameras for wildfire detection and monitoring systems entails an adequate understanding of the application requirements and capabilities of each device, which change depending on manufacturers and camera models. Furthermore, considering UAV-based applications, the integration of this type of device in robotic perception frameworks is not straightforward, requiring knowledge of the underlying image processing algorithms, which are highly nonlinear. For this reason, based on insights gained from the comprehensive analyses presented, several remarks deserve to be highlighted.

This work explored a series of automatic gain control methods available in the firmware and software of thermal cameras, which are based on histogram-based algorithms for data compression and contrast enhancement. In particular, testing the applicability of several alternatives in fire scenarios, the results revealed that advanced contrast enhancement techniques degrade the ability to distinguish the fire from the surroundings, preventing an accurate identification of the burning area. Indeed, the linear approach registered the best results, as identified in Figure 7.

Since histogram-based algorithms promote highly nonlinear effects in image data, color-based features require careful interpretation because tracking the hottest areas can be misleading depending on the scene context. In that sense, extending state-of-the-art color-based approaches [22] by also including raw-based data provides enhanced situation awareness, thus potentially enabling improvements in robotic perception.

By testing the proposed method in distinct scenarios, under static image acquisition conditions, and also from onboard mobile platforms (e.g., a tethered helium balloon), it was possible to validate the approach in both contexts. Nonetheless, for integration in robotic platforms, it is advisable to employ means of camera stabilization to minimize the effect of the external disturbances due to the movement of the platform and consequent changes in the field of view of the camera.

In turn, the operating distance conditions of these sensors play an important role when applying heuristic methods and, as such, the performance shall be fine-tuned depending on the image capture setup. Moreover, the characterization of the device in terms of sensor response is fundamental for this type of application because although manufacturers define nominal operating conditions, since fire is a source of extreme temperatures, it is possible to successfully detect fire at long distances as well, since a high signal-to-noise ratio is preserved.

Regarding the integration in robotic perception pipelines for autonomous navigation of UAVs, it was also verified that the thermal sensitivity of these sensors is also able to convey depth perception. However, in order to apply the methodology proposed in this work, the camera model has to provide access to the raw data on-line so that it can be incorporated and used in the image processing pipeline of the robotic perception framework.

## 6. Conclusions

The study conducted in this work provides a comprehensive introduction to adapting thermal imaging sensors for wildfire detecting and monitoring systems. Since these phenomena are increasing in intensity and frequency, there is increased urgency in the early detection, monitoring and surveillance of these events. For these reasons, leveraging mobile robotics in this type of extreme environment requires devising of application-specific techniques.

For that purpose, this paper explored the foundations of image processing of thermal imaging, covering the most used automatic gain control methods and the color mapping schemas responsible for generating thermal images encoded in pseudocolor.

The proposed data-driven feature engineering approach, combining expert knowledge about thermal imaging sensors as well as color-based segmentation heuristics, offers improvements in situation awareness in comparison to existing methods focusing only on one data type.

The study of the response of thermal imaging sensors in fire scenarios as well as situations without fire considering raw sensor data and color-based features was revealed to be fundamental for providing a better intuition of the working mechanisms of these sensors and the expected behavior under such conditions. By evaluating how the features evolve over time, these analyses provided a clear understanding of the nonlinear adaptive color scale, providing important insights relative to the sensing capabilities of commercial off-the-shelf thermal cameras, and thus establishing a solid foundation for the development of robotic perception algorithms.

Considering that wildfire detection and monitoring are safety-critical applications, there is a great benefit in exploring highly interpretable feature engineering approaches in order to subsequently combine these methods with intelligent soft sensor approaches, enabling better model understanding and interpretation.

## Figures and Tables

**Table 1:** Summary of the specifications of the thermal cameras.

	FLIR SC660	FLIR Vue Pro
Spatial resolution (px)	640 × 480	336 × 256
Temporal resolution (Hz)	30	8.3
Bit resolution (bit)	14	16
Focal length (mm)	19	9.0
Horizontal field of view (FOV) (°)	45	35
Vertical FOV (°)	34	27
Spectral band ($10^{-6}$ m)	7.5∼13.5	7.5∼13.5
Measurement range (°C)	−40∼+1500	−60∼+150
Size (L × W × H) (mm)	299 × 144 × 147	63 × 44.4 × 44.4
Weight (g)	1800	92.1–113.4

**Table 2:** RGB segmentation thresholds for feature construction [22].

Features	Gray			Green			Red		
channels	R	G	B	R	G	B	R	G	B
upper limit	253	199	185	143	169	157	255	73	71
lower limit	149	171	160	98	90	86	103	89	85
